# Molecular mechanisms and application potential of MYC2 transcription factor-mediated plant stress responses

**DOI:** 10.3389/fpls.2026.1943016

**Published:** 2026-10-05

**Authors:** Liu-Qing Yang, Si-Lin Shu, Yong-Hao Zhang, Gang Huang

**Affiliations:** 1 Qiandongnan Nationalities Polytechnic, Kaili, Guizhou, China; 2 Collaborative Innovation Center for Breeding and Application Research of Characteristic Plant Seedlings, Kaili, Guizhou, China

**Keywords:** abiotic stress, biotic stress, hormone crosstalk, jasmonic acid signalling, MYC2 transcription factor, secondary metabolism

## Abstract

Plant adaptation to drought, salinity, temperature extremes, pathogens, and herbivorous insects depends on coordinated hormone signalling, transcriptional control, and metabolic reprogramming. The jasmonic acid (JA) pathway is a major regulator of defence and the growth-defence trade-off, and the bHLH transcription factor MYC2 is a central node in JA output. Mechanistically, MYC2 is controlled through demonstrated protein-protein interactions with JAZ repressors, MED25, and selected transcriptional or chromatin regulators; it binds G-box-related cis-elements to regulate specific promoters, whereas many reported links to LOX/AOS, CBF, and antioxidant pathways are transcriptional or pathway-level rather than physical interactions. This integrative narrative review compares MYC2 orthologues across species, distinguishes MYC2-specific functions from those shared with MYC3/MYC4 or other clade IIIe bHLHs, and organises beneficial and detrimental stress phenotypes around pathogen lifestyle, target-gene polarity, tissue and developmental context, paralogue redundancy, and experimental design. We integrate genome-wide DNA-binding, transcriptomic, metabolomic, and protein-level studies, and critically evaluate demonstrated versus prospective applications of CRISPR/Cas genome editing. The synthesis identifies regulatory strategies that may exploit MYC2 networks whilst limiting growth, defence, and quality trade-offs.

## 1 Introduction

Plants in natural environments are often exposed simultaneously to drought, salinity, low and high temperatures, and pathogen infection. Their adaptive responses depend on the coordinated integration of signalling pathways, transcriptional regulation, hormone regulation, and metabolic reprogramming (Suzuki et al., 2014). Transcription factors connect external stimuli to downstream gene-expression reprogramming. MYC transcription factors belong to the bHLH family and constitute an important transcriptional output of the jasmonic acid (JA) signalling pathway (Kazan and Manners, 2013; Lian et al., 2017).

Amongst MYC proteins, MYC2 is the most extensively studied regulator of JA signalling. *Arabidopsis JASMONATE-INSENSITIVE1*/*MYC2* was initially shown to discriminate between JA-dependent defence branches. Subsequent work demonstrated promoter-dependent activation and repression by MYC2 and additive functions with MYC3 and MYC4 (Lorenzo et al., 2004; Dombrecht et al., 2007; Fernández-Calvo et al., 2011). Genome-wide studies have since expanded the MYC2 target space beyond canonical JA genes. Joint DAP-seq and RNA-seq in maize identified thousands of candidate ZmMYC2 targets, whereas time-resolved RNA-seq, ChIP-seq, and metabolomics in rice resolved core, tissue-specific, and negative-feedback sectors of JA defence (Chen et al., 2024; Liu et al., 2024). A water-spray time course in *Arabidopsis* identified 266 transient MYC2/MYC3/MYC4-dependent genes, including an 82-gene core regulon. The same clade amplified JA and JA-Ile accumulation through positive feedback involving JA-biosynthetic genes (Van Moerkercke et al., 2019). Integrated transcriptomic and (phospho)proteomic analyses in *Arabidopsis* linked MYC2/MYC3/MYC4-dependent transcription to protein abundance, phosphorylation, alternative splicing, and chromatin remodelling (Zander et al., 2020).

Previous reviews established MYC2 as a central JA regulator (Kazan and Manners, 2013), summarised its roles in transcriptional reprogramming and stress tolerance (Song et al., 2022), and examined its physiological and specialised-metabolism functions (Luo et al., 2023; Zeng et al., 2025). The present review extends this work by separating physical interaction, promoter occupancy, transcriptional regulation, and pathway association. It also distinguishes orthologue-specific functions from MYC2/MYC3/MYC4-redundant effects across species. Contrasting beneficial and detrimental phenotypes are analysed in relation to hormone crosstalk and growth-defence allocation. Finally, demonstrated genome-editing outcomes are evaluated separately from prospective precision-engineering strategies.

Accordingly, this article is an integrative narrative review rather than a systematic review. We prioritise genetic and mechanistic studies that explicitly connect a MYC2 orthologue to a molecular target, interacting partner, or stress phenotype, and use recent multi-omics and genome-editing studies to identify translational opportunities and evidence gaps. Comparative tables summarise the strength and type of evidence so that direct mechanisms are not conflated with transcriptional or pathway-level associations. **Figure 1** provides the overall scope of the review.

**Figure 1:**
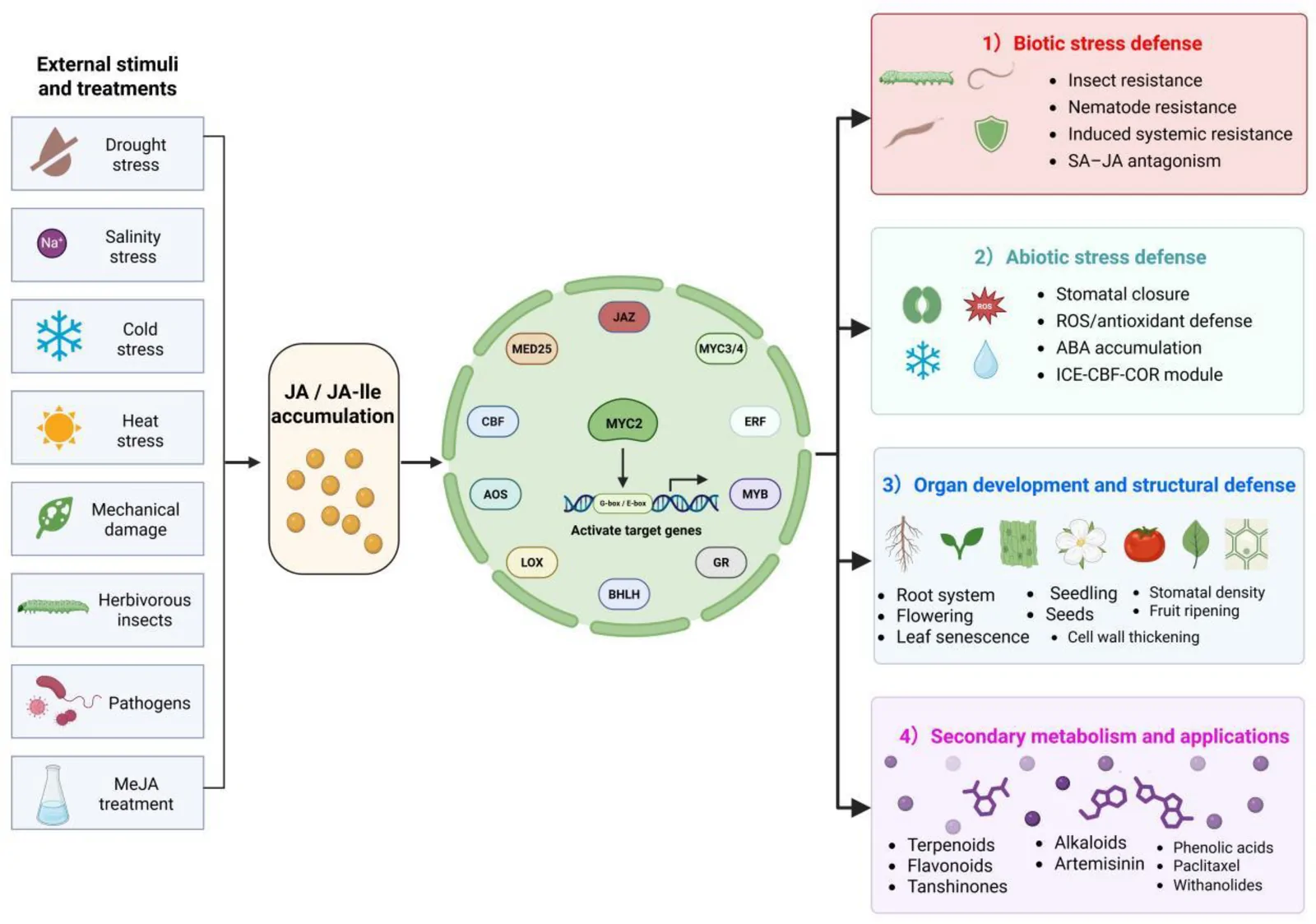
Overview of MYC2-mediated plant stress responses and biological functions. External stimuli and MeJA treatment promote JA/JA-Ile accumulation and relieve JAZ-mediated repression of MYC2. MYC2 binds G-box-related elements and is connected to transcriptional regulators, stress responses, development, structural defence, and secondary metabolism. Arrows in this overview denote regulatory flow and do not by themselves imply direct protein-protein interaction or promoter binding; evidence classes for representative links are specified in **Table 1**. JA, jasmonic acid; JA-Ile, jasmonoyl-L-isoleucine; MeJA, methyl jasmonate. Created with BioRender.com.

## 2 Structural features of MYC2

MYC2 belongs to the bHLH protein family. Its N terminus contains a JAZ-interaction domain (JID) and a transcriptional activation domain (TAD), whereas the C-terminal bHLH domain mediates DNA binding and dimerisation. The JID binds JAZ proteins, and the TAD recruits co-activators such as MED25. The basic region recognises the G-box (5′-CACGTG-3′) and related promoter elements (Shoji and Hashimoto, 2011; Song et al., 2022). *Arabidopsis* MYC2 can form tetramers that enhance DNA binding (Lian et al., 2017). MYC2-related bHLHs also recognise conserved A/T-rich flanking modules and associated DNA-shape features (López-Vidriero et al., 2021). Structural studies, conducted mainly with MYC3, showed that JAZ proteins bind a conserved N-terminal region and compete with MED25. Conservation of this interface suggests that the mechanism also applies to MYC2 (Zhang et al., 2015). Genome-wide mapping of ZmMYC2 binding sites further indicated that its candidate targets span JA responses, development, and stress adaptation (Liu et al., 2024). Domain architecture, oligomerisation, JAZ binding, and cis-element context therefore shape the MYC2 target repertoire. **Figure 2** summarises this architecture and the JA-dependent derepression mechanism.

**Figure 2:**
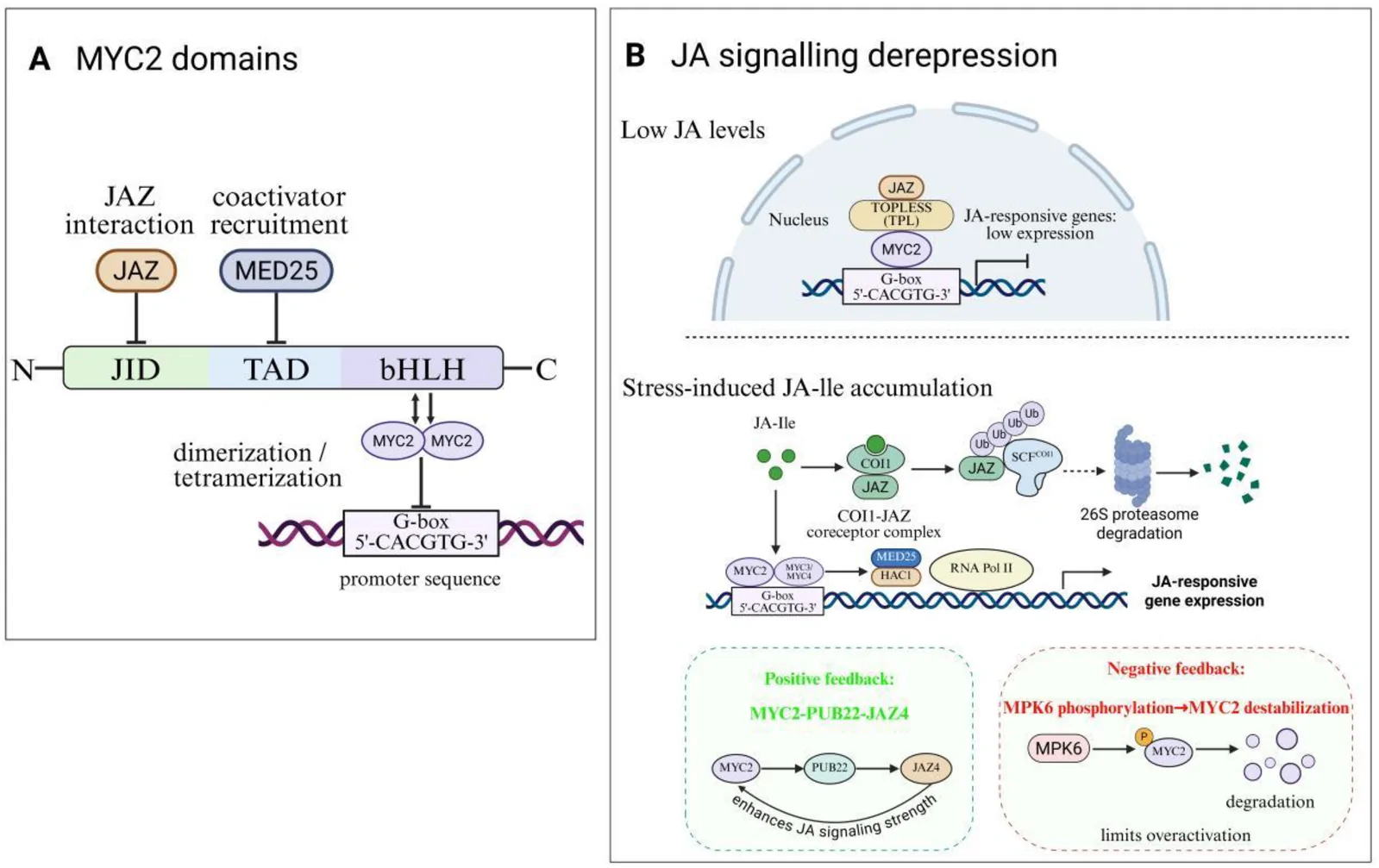
MYC2 domains and JA-dependent transcriptional derepression. **(A)** The JAZ-interaction domain (JID), transcriptional activation domain (TAD), and bHLH domain mediate JAZ binding, co-activator recruitment, oligomerisation, and G-box recognition, respectively. **(B)** JA-Ile promotes COI1-dependent JAZ degradation, releasing MYC2 to recruit MED25, HAC1, and RNA polymerase II. Solid connectors explicitly labelled as binding or recruitment denote demonstrated molecular interactions; dashed connectors denote degradation or indirect regulatory transitions. The MYC2-PUB22-JAZ4 and MPK6-MYC2 modules provide positive and negative feedback, respectively. Created with BioRender.com.

## 3 MYC2-mediated regulation of JA signalling

JA and its derivatives (JAs) are oxylipin signalling molecules that rapidly accumulate after mechanical damage, herbivore feeding, infection by necrotrophic pathogens, and various abiotic stresses. They coordinate defence, metabolic regulation, and growth and development (Breeze, 2019; Luo et al., 2023). At low JA levels, JAZ proteins bind MYC2 and recruit TOPLESS/TPL-related co-repressors, thereby suppressing MYC2-mediated transcriptional activation (Shyu et al., 2012; Kazan and Manners, 2013). Stress-induced accumulation of JA-Ile promotes formation of the COI1–JAZ co-receptor complex (Fonseca et al., 2009; Sheard et al., 2010). JAZ proteins are then ubiquitinated by SCF^COI1 and degraded by the 26S proteasome, releasing MYC2 from repression (Thines et al., 2007). MYC2 and its homologues MYC3 and MYC4 can subsequently bind G-box and related cis-elements and activate JA-responsive genes (Nagels Durand et al., 2016). Through MED25, MYC2 promotes assembly of the transcription-initiation complex and recruitment of RNA polymerase II. In cooperation with chromatin regulators such as HAC1, it also modulates histone modifications at target promoters and enhances transcription of JA-responsive genes (You et al., 2019; Zhai and Li, 2019). Because JAZ proteins can competitively influence formation of the MYC2–MED25 complex, release from JA repression reflects not only JAZ degradation but also reassembly of the MYC2 co-activator complex (Zhang et al., 2015). Recent studies have further revealed dynamic control of MYC2. In tomato, the MYC2–PUB22–JAZ4 module forms a positive-feedback loop in JA signalling and influences JAZ stability and signal strength (Wu et al., 2024). These findings underscore the importance of feedback control in maintaining appropriate JA signal strength. MYC2-mediated JA output is therefore generated by the combined action of JAZ degradation, MED25-dependent transcriptional activation, chromatin regulation, and control of protein stability.

Mechanistic statements about MYC2 require an explicit evidence hierarchy. In this review, protein-protein interaction is reserved for physical association supported by assays such as co-immunoprecipitation, pull-down, yeast two-hybrid, or bimolecular fluorescence complementation. Direct transcriptional regulation requires promoter occupancy or DNA binding (for example, ChIP, EMSA, yeast one-hybrid, or DAP-seq) together with expression or reporter evidence when available. Changes in transcript abundance without promoter evidence are described as transcriptional regulation, whereas genetic dependence, hormone responses, or correlated expression are described as pathway-level associations. Genome-wide DAP-seq identifies *in vitro* occupancy and therefore defines candidate direct targets; it is not equivalent to *in vivo* ChIP plus genetic validation. These categories are used throughout **Table 1**.

**Table 1:** Cross-species comparison of MYC2 stress functions and supporting evidence.

Species/orthologue	Stress or trait	Target or partner and direction	Evidence type/methods	Net phenotype and context	Reference
*Arabidopsis*/AtMYC2	Salt stress	Represses *CAT2* expression; direct DNA binding not established	*myc2* and *myc2 cat2* genetics; qPCR; ROS and catalase assays	Higher MYC2 output lowers seedling salt tolerance through excess H2O2	Song et al., 2021
*Arabidopsis*/AtMYC2	*P. syringae*	Directly activates *ZAT18*; *ZAT18* represses *EDS1*	Promoter-binding assays; *zat18* CRISPR; epistasis; SA measurement	Facilitates infection by weakening SA immunity	Gao et al., 2022
*Arabidopsis*/MYC2/3/4	Herbivory/glucosinolates	Cooperates with glucosinolate MYBs	Higher-order mutants; expression and metabolite profiling; insect assays	Clade-redundant positive defence; not MYC2 specific	Schweizer et al., 2013
Tomato/SlMYC2	Drought	Directly represses *SlPP2C1* and *SlRR26*	Guard-cell transcriptomics; promoter assays; transgenics and CRISPR *slrr26*	Promotes ROS-dependent stomatal closure and drought tolerance	Zhao et al., 2023
Tomato/SlMYC2	Drought	Directly represses *SlCHS1*	DNA-binding/reporter assays; guard-cell metabolites, ROS, stomata	Promotes closure; illustrates beneficial local ROS signalling	Xu et al., 2022
Sorghum/SbMYC2	Drought	Directly activates *SbGR1*; binds SbJAZ proteins	OE and VIGS; Y1H/EMSA/LUC; Y2H; ROS and physiology	Improves antioxidant capacity and drought tolerance	Wang et al., 2024
Poplar/PpnMYC2	Stomatal density/drought	Activates *EPF2*/*EPFL4*; represses *EPFL9* directly	RNA-seq; Y1H; EMSA; ChIP-qPCR; LUC; OE plants	Reduces stomatal density; increases WUE and drought resistance	Xia et al., 2024
Rice/OsMYC2	Brown planthopper	Initiates *JAMYB* and bHLH6 transcriptional cascades	ChIP-seq/RNA-seq/metabolomics; genetics; promoter assays	Enhances direct and indirect (volatile-mediated) defence with feedback control	Chen et al., 2024; Liu et al., 2025
Maize/ZmMYC2	JA/stress network	Binds thousands of candidate target loci	DAP-seq plus RNA-seq overlap	Broad candidate network; *in vivo* causality remains to be tested	Liu et al., 2024
Tea/CsMYC2	Osmotic stress	Transcriptionally activates *CsLOX7*/*CsAOS2* feedback	Promoter-binding/reporters; expression; enzyme and stress phenotyping	Promotes JA amplification and osmotic adaptation	Zhu et al., 2024
Tobacco/NtMYC2a	Bacterial wilt	Supports *NtJAR1*/*NtCOI1* pathway expression; direct targets unresolved	CRISPR knockout; RNA-seq; pathogen, agronomic, and chemical phenotyping	Knockout narrows leaves and decreases resistance	Xiao et al., 2024
Oilseed rape/*BnaA05.MYC2*	Clubroot	Directly represses *BnaA07.SUC2*	Y1H; EMSA; LUC; OE/complementation; CRISPR target knockout	Restricts sucrose supply to pathogen and enhances resistance	Liu et al., 2026

Evidence terminology: PPI, demonstrated protein-protein interaction; direct promoter regulation, DNA occupancy/binding plus transcriptional evidence; transcriptional regulation, expression changes without direct promoter-binding evidence.

MYC2 also belongs to a functionally redundant clade. *Arabidopsis* MYC3 and MYC4 are JAZ-interacting bHLHs that act additively with MYC2, and many strong phenotypes emerge most clearly in *myc2 myc3 myc4* higher-order mutants (Fernández-Calvo et al., 2011). Glucosinolate accumulation and herbivore defence, seed storage-protein accumulation, JA-induced senescence, and flowering inhibition are therefore clade-level outputs unless a study resolves a MYC2-specific contribution (Schweizer et al., 2013; Qi et al., 2015b; Gao et al., 2016; Wang et al., 2017). Redundancy is also lineage dependent: tomato MYC1 and MYC2 jointly control constitutive and inducible specialised metabolism, *Salvia miltiorrhiza* SmMYC2a and SmMYC2b have related but non-interchangeable functions, and rice OsMYC2 can cooperate with MYC2-like proteins (Zhou et al., 2016; Ogawa et al., 2017; Swinnen et al., 2022). Accordingly, we attribute a function specifically to MYC2 only when single-gene genetics, direct target evidence, or complementation supports that assignment; otherwise, we use MYC2/MYC3/MYC4 or MYC-clade terminology.

## 4 MYC2 in hormone crosstalk and stress responses

Although MYC2 is a major regulator of JA output, it also cross-regulates ABA, GA, ET, SA, and IAA pathways. This crosstalk connects stress perception with transcription, defence metabolism, and growth. During transitions between growth and flg22-induced pattern-triggered immunity, targeted turnover profiling of 99 proteins identified MYC2/MYC3/MYC4-dependent changes in tryptophan and glucosinolate biosynthesis, IAA transport, and photosynthetic proteins (Abukhalaf et al., 2023). These findings extend MYC-clade regulation beyond transcript abundance. The direction of MYC2 activity depends on stress type, tissue state, interacting proteins, and downstream targets.

### 4.1 Hormone crosstalk and the growth–defence trade-off

MYC2 was first shown to activate ABA-responsive transcription and *rd22* expression, placing it at the JA-ABA interface (Abe et al., 2003). The ABA receptor PYL6/RCAR9 physically interacts with MYC2, and ABA enhances this association; promoter and reporter analyses linked this interaction to MYC2 activity at *JAZ6* and *JAZ8* (Aleman et al., 2016). MED25 physically associates with MYC2 to support JA-responsive transcription but negatively modulates ABI5-dependent ABA responses (Chen et al., 2012). In GA signalling, DELLA proteins bind JAZ repressors and can release MYC activity by competition for JAZ association (Hou et al., 2010); MYC2 can also physically interact with DELLA proteins in the regulation of sesquiterpene synthase genes (Hong et al., 2012). Complementing this protein-level crosstalk, JA lowers bioactive GA by repressing GA-biosynthetic genes and inducing *GA2ox* in a MYC2/3/4-dependent manner; miR5998-mediated post-transcriptional control of *GA3ox1* further links MYC2 activity to growth inhibition (Fukazawa et al., 2023). In the ET pathway, MYC2 physically interacts with EIN3 and suppresses EIN3 transcriptional activity, thereby modulating JA-ET antagonism (Song et al., 2014). These molecular interfaces show that hormone crosstalk can alter MYC2 partner availability as well as the identity and polarity of downstream targets. By contrast, associations between MYC2 and broader ABA, CBF, LOX/AOS, or antioxidant outputs should be described as direct promoter regulation only when DNA-binding evidence is available, and otherwise as transcriptional or pathway-level regulation. **Figure 3** summarises the major regulatory relationships through which MYC2 integrates JA signalling with ABA, ET, SA, and GA pathways during plant stress responses.

**Figure 3:**
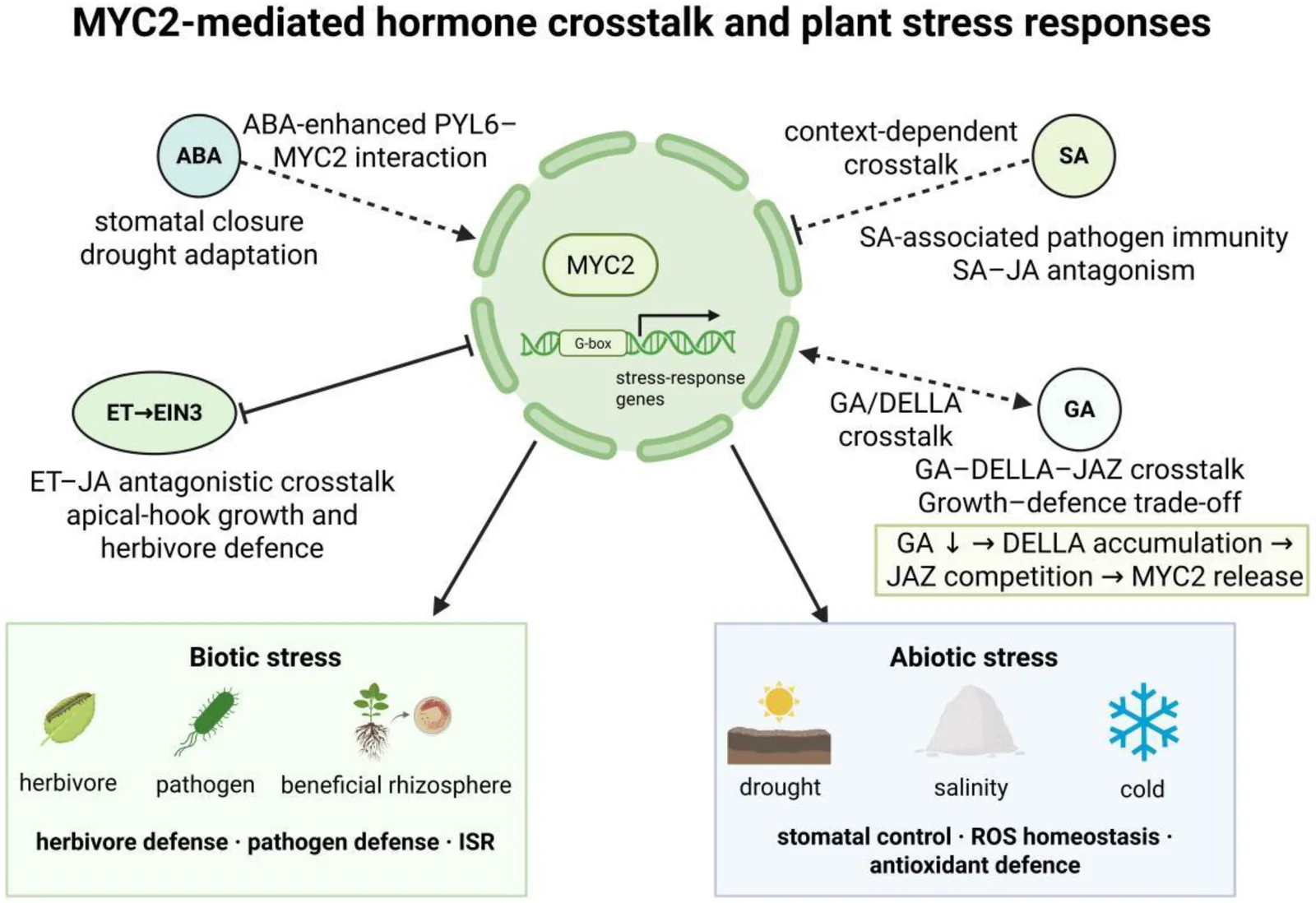
MYC2-mediated hormone crosstalk in plant stress responses. MYC2 integrates JA signalling with ABA, ET, SA, and GA pathways. Solid arrows denote supported regulatory direction, dashed lines denote indirect or context-dependent relationships, and blunt-ended lines indicate antagonistic regulation. A line labelled as interaction refers to demonstrated physical association; other lines should not be interpreted as protein-protein interaction. ISR, induced systemic resistance. Created with BioRender.com.

### 4.2 Interactions with transcription factors and multilayer regulatory networks

MYC2 networks combine physical complexes with hierarchical transcriptional cascades. At the core JA node, JAZ repressors bind MYC2, whereas MED25, HAC1, and LEUNIG_HOMOLOG assemble a co-activator and chromatin-regulatory complex; MPK6 phosphorylation changes MYC2 stability, and the tomato histone demethylase JMJC3 physically associates with MYC2 during thermotolerance (You et al., 2019; Im et al., 2025; Xu et al., 2026). At a second layer, MYC2 orthologues directly regulate intermediary transcription-factor genes that initiate downstream cascades. Demonstrated examples include *MYB46* in *Arabidopsis* (Im et al., 2024), *JAMYB* in rice (Liu et al., 2025), *SlMYB12* in tomato (Deng et al., 2025), *ORCA* genes in *Catharanthus roseus* (Zhang et al., 2011), and *MTB1–MTB3* in tomato (Liu et al., 2019). These findings support promoter-level transcriptional cascades; they do not imply that MYC2 physically interacts with every terminal enzyme or stress-response protein. A third layer comprises physical or competitive interactions with regulators such as EIN3 and CrGBF1/2, which can redirect promoter selection or attenuate transactivation (Song et al., 2014; Sui et al., 2018). The resulting network contains positive amplification, negative feedback, and branch competition, explaining why MYC2 activity cannot be represented as a single linear JA pathway.

Network composition is species and tissue specific. Rice multi-omics resolved an early, cross-tissue MYC2-bHLH6 core sector, late tissue-specific metabolic sectors, and NAC/JAZ/catabolic negative-feedback sectors (Chen et al., 2024). In tomato, MYC2-MED25 activates the *SlMYB12* transcriptional cascade in fruit, whereas in *Catharanthus roseus*, MYC2-induced GBFs can antagonise MYC2 at shared T/G-boxes and through dimerisation (Sui et al., 2018; Deng et al., 2025). These examples distinguish two often conflated relationships: MYC2 can physically associate with a regulator, or it can transcriptionally induce that regulator and thereby act indirectly on downstream genes.

### 4.3 Adaptation to biotic stress

During herbivore feeding, mechanical damage, and rhizobacteria-induced resistance, MYC2 or the MYC2/3/4 clade often promotes JA-dependent defence. *Arabidopsis* MYC2 contributes to rhizobacteria-induced systemic resistance and defence priming (Pozo et al., 2008), whereas the rice MYC2-*JAMYB* transcriptional cascade controls phenylpropanoid and volatile-associated genes that enhance brown-planthopper resistance (Liu et al., 2025). A complementary rice study linked MYC2 to a MKKK55/62/70–MKK3–MPK7/14 MAPK cascade that increases cellulose deposition in sclerenchyma cell walls and strengthens structural resistance to brown planthoppers (Wu et al., 2025). Oviposition can alter this JA output before feeding begins: *Pieris brassicae* eggs trigger local salicylic acid accumulation, suppress JA-responsive gene induction, and increase the growth of the generalist *Spodoptera littoralis*; this illustrates SA–JA antagonism at the egg stage rather than direct MYC2 regulation (Bruessow et al., 2010). Not every JA-defence mechanism, however, is MYC2 mediated. In tomato root-knot nematode infection, ATG8-dependent degradation of JAM1 releases an ERF1-MED25 branch and activates JA-defence genes (Zou et al., 2023); because that study did not establish a requirement for MYC2, it is included here as a comparator showing branch allocation within JA signalling, not as evidence of direct MYC2 action. Context can also reverse the apparent benefit of MYC2. During *Pseudomonas syringae* infection, MYC2 directly activates *ZAT18*, which represses *EDS1* and SA accumulation and thereby facilitates bacterial infection (Gao et al., 2022). *Populus* MYC2 paralogues similarly restrict colonisation by the beneficial ectomycorrhizal fungus *Laccaria bicolor* by reprogramming root defence and terpene metabolism (Marqués-Gálvez et al., 2024). Thus, the net phenotype depends on attacker lifestyle, JA-SA balance, and whether microbial colonisation is pathogenic or mutualistic.

### 4.4 Adaptation to abiotic stress

Under drought and osmotic stress, MYC2 often promotes adaptation by coordinating stomatal movement, hormone crosstalk, JA feedback, and antioxidant capacity. Tomato SlMYC2 directly represses the negative regulators *SlPP2C1* and *SlRR26*, increasing JA-dependent stomatal closure (Zhao et al., 2023), and directly represses *SlCHS1* in guard cells, lowering flavonol buffering and permitting a ROS signal that promotes closure (Xu et al., 2022). Oriental-melon CmMYC2 binds the *CmLOX10* promoter, forming a transcriptional feedback loop that supports JA accumulation and stomatal closure (Xing et al., 2020). Sorghum SbMYC2 directly activates *SbGR1* and increases glutathione-dependent ROS control (Wang et al., 2024). In wheat, drought-responsive transcriptomics and promoter analyses identified MYC2 upstream of the melatonin-biosynthetic gene *ASMT*; coordinated MYC2–*ASMT* expression and melatonin-associated antioxidant and photosynthetic traits support a drought-tolerance module, although direct promoter occupancy remains to be demonstrated (Shamloo-Dashtpagerdi et al., 2023). In tea, CsMYC2-dependent promoter regulation of *CsLOX7*/*CsAOS2* forms a JA-biosynthetic feedback loop; this is a transcriptional circuit, not a physical interaction between MYC2 and LOX/AOS enzymes (Zhu et al., 2024). These mechanisms show that ROS can be either a spatially restricted signal for stomatal closure or a damaging systemic load, depending on tissue and duration.

Temperature and salinity responses provide a second contrast. Tomato MYC2 promotes *NCED2* transcription and cold tolerance through ABA accumulation (Ding et al., 2022), whereas heterologous *TaMYC2* overexpression in *Arabidopsis* is associated with increased ICE-CBF-COR pathway expression and freezing tolerance; the latter is pathway-level evidence and does not establish direct TaMYC2 binding to CBF genes (Wang et al., 2022). In a related heterologous assay, Caucasian-clover *TaMYC2* overexpression in tobacco improved drought and cold tolerance, lowered lipid peroxidation, and increased SOD, CAT, POD, proline, and stress-responsive gene activity; this supports an antioxidant-associated phenotype but not a resolved direct target mechanism (Zhao et al., 2022). *SmMYC2* overexpression enhances salt tolerance in *Arabidopsis* and *Salvia miltiorrhiza* hairy roots (Deng et al., 2023). Conversely, in *Arabidopsis* seedlings, JA-MYC2 represses *CAT2* expression without reported direct promoter-binding evidence, elevates H2O2, and reduces salt tolerance (Song et al., 2021). These opposite outcomes caution against transferring a phenotype across species or equating heterologous overexpression with native-gene function.

MYC2 phenotypes depend more on regulatory context than on stress category. Activation of antioxidant capacity, ABA-supported stomatal closure, structural defence, or stress-matched metabolites can be protective. Detrimental effects arise when MYC2 represses antioxidant genes, strengthens JA-SA antagonism against biotrophic or hemibiotrophic pathogens, restricts beneficial symbionts, or imposes excessive growth costs. **Table 1** compares these mechanisms and their evidence types across species.

### 4.5 Cross-species conservation, divergence, and determinants of opposite outcomes

Five variables account for much of the reported cross-species divergence: attacker lifestyle, target-gene polarity, paralogue dosage, biological context, and experimental design. Attacker lifestyle changes the relative value of JA, SA, and ET signalling. Activation of *SbGR1* or repression of ABA negative regulators is protective, whereas repression of *CAT2* or activation of *ZAT18* can be detrimental. Paralogue number determines whether single-gene perturbation is buffered, especially in polyploids and species with several MYC2-like genes. Tissue, developmental stage, and stress duration also determine whether ROS production, stomatal closure, senescence, or metabolite accumulation is adaptive. Finally, constitutive or heterologous overexpression, native knockout, acute treatment, and chronic stress do not provide interchangeable evidence. Seedling phenotypes should likewise not be extrapolated directly to yield or field performance.

These variables also interact. A transcriptional circuit that increases defence in a leaf sheath may reduce reproductive performance if constitutively activated, and a metabolite that deters herbivores may compromise food quality or divert carbon and nitrogen. Accordingly, the most informative studies combine native-gene genetics, direct target assays, tissue-resolved profiling, and measurements of both stress performance and growth or quality. Cross-species comparisons should therefore report the orthologue and paralogues examined, tissue and developmental stage, perturbation type, stress regime, directness of molecular evidence, and both beneficial and adverse phenotypes.

## 5 MYC2 in growth and development

MYC2 is activated not only under stress to initiate defence transcriptional programmes but also to regulate JA-mediated growth, development, and defence. Its functions often overlap or add to those of homologous bHLH transcription factors such as MYC3 and MYC4 and are controlled by JAZ repressors. Once JA signalling is activated, JAZ proteins are degraded, releasing MYC transcription factors and initiating downstream transcriptional responses (Fernández-Calvo et al., 2011).

### 5.1 Root and seedling development

In the *Arabidopsis* root meristem, MYC2 directly represses *PLT1* and *PLT2*, linking JA signalling to the auxin-dependent root stem-cell network (Chen et al., 2011). During adventitious-root formation, MYC2-dependent JA signalling represses *CKX1* and activates *RAP2.6L*, altering cytokinin homeostasis (Dob et al., 2021). In rice, OsMED25 physically interacts with OsMYC2 and is required for JA-mediated root development and leaf senescence, supporting partial conservation of the co-activator mechanism in monocots (Suzuki et al., 2021). MYC2 physically interacts with EIN3 to antagonise ethylene-promoted apical-hook formation (Zhang et al., 2014) and genetically and functionally cooperates with HY5 during light-regulated seedling morphogenesis (Chakraborty et al., 2019). SUMO conjugation of MYC2 provides an additional post-translational layer in blue-light responses (Srivastava et al., 2022).

### 5.2 Shoot and reproductive development

In poplar, PpnMYC2 directly activates *EPF2*/*EPFL4* and represses *EPFL9*, reducing stomatal density and improving water-use efficiency (Xia et al., 2024). In *Arabidopsis* seed and reproductive development, MYC2, MYC3, and MYC4 act redundantly or additively: higher-order mutants reveal roles in seed production, storage-protein accumulation, JA-mediated flowering inhibition, and MYB21/MYB24-containing complexes that control stamen maturation (Qi et al., 2015a; Gao et al., 2016; Wang et al., 2017). These outputs should therefore not be attributed to MYC2 alone. During apple fruit ripening, MdMYC2 directly binds and activates *MdACS1*, *MdACO1*, and *MdERF3* promoters; it also physically interacts with the repressor MdERF2, preventing MdERF2 from blocking *MdERF3* and *MdACS1* (Li et al., 2017).

### 5.3 Senescence, cell walls, and structural defence

During leaf senescence, MYC2, MYC3, and MYC4 antagonise bHLH subgroup IIId factors and promote JA-induced senescence by activating senescence-associated genes such as *SAG29* (Qi et al., 2015b). In rice, OsMED25 participates in OsMYC2-mediated leaf senescence, further showing that MYC2-dependent senescence regulation is not restricted to *Arabidopsis* (Suzuki et al., 2021). Recent work also showed that JA activates secondary cell-wall biosynthesis through the MYC2–*MYB46* module, placing MYC2 in wall thickening, lignification, and structural defence (Im et al., 2024). MYC2 therefore does not simply promote or inhibit organ growth. Instead, it allocates resources amongst cell division, differentiation, maturation, senescence, and structural reinforcement across organs and developmental stages.

Overall, MYC2 and its homologues participate in root formation, seedling morphogenesis, stomatal development, reproductive-organ maturation, fruit ripening, leaf senescence, and cell-wall formation through crosstalk between JA and multiple hormonal and environmental signals. Future research should distinguish MYC2 functions across tissues, developmental stages, and species, particularly avoiding direct extrapolation of conclusions from *Arabidopsis* to crops, forest trees, and medicinal plants. **Figure 4** summarises these developmental functions.

**Figure 4:**
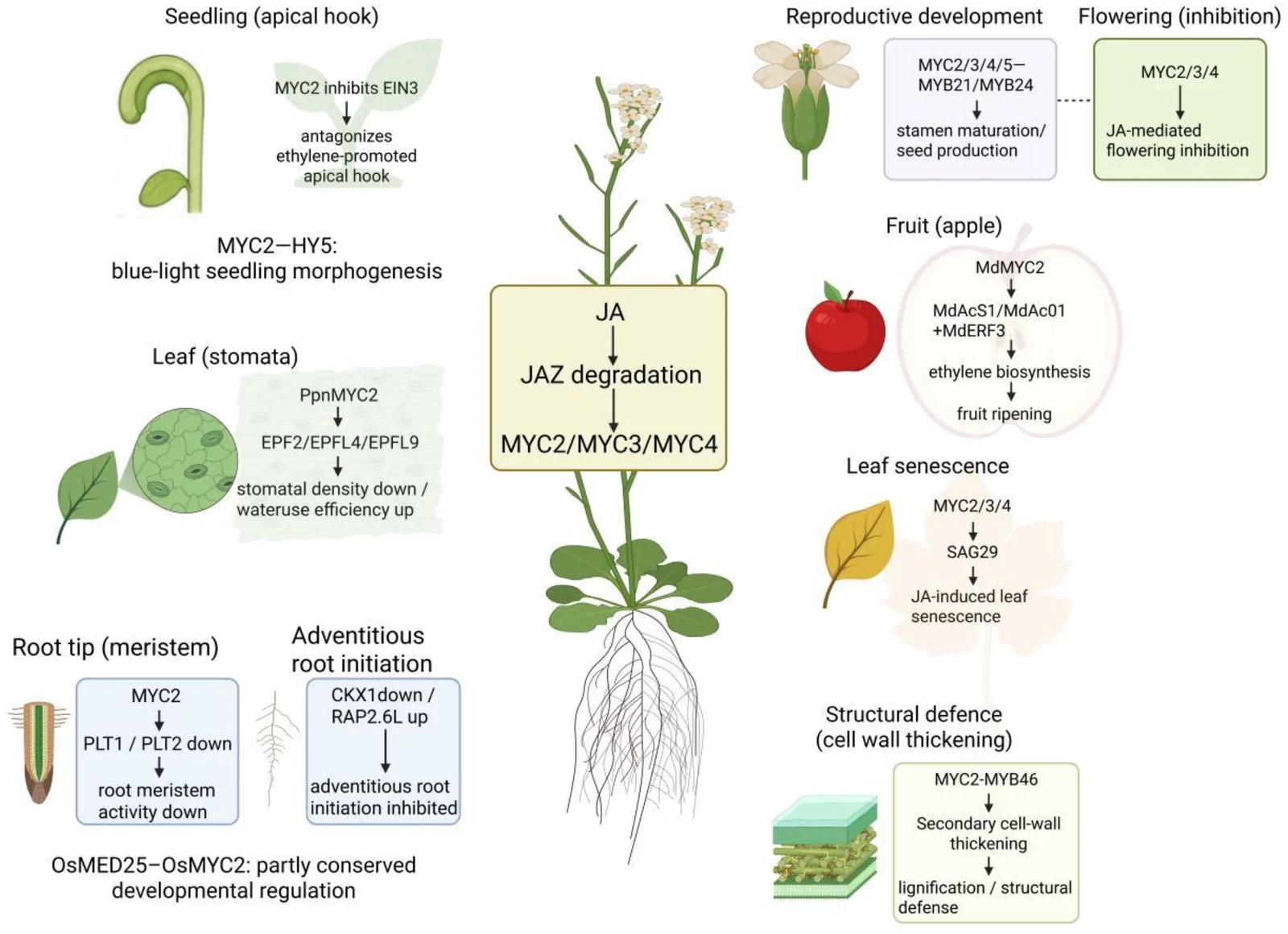
Roles of MYC2 in plant growth, development, senescence, and structural defence. JA-induced JAZ degradation releases MYC2 and related MYC proteins to regulate seedling morphogenesis, stomatal development, root growth, reproduction, fruit ripening, senescence, and secondary-cell-wall formation. Solid arrows show regulatory direction; dashed lines indicate indirect, context-dependent, or clade-redundant relationships. Only connectors explicitly described as binding or interaction denote direct molecular evidence. *PLT1*/*2*, *CKX1*, *RAP2.6L*, *MdACS1*, *MdACO1*, and *SAG29* are representative targets or downstream genes. Created with BioRender.com.

## 6 MYC2 and secondary metabolism

The central role of MYC2 in secondary metabolism is to convert JA signalling and external stimuli, including injury, herbivore feeding, pathogen infection, and methyl jasmonate (MeJA) treatment, into transcriptional activation of structural genes in metabolic pathways. Unlike regulation of a single enzyme gene, MYC2 usually acts upstream of the signal–transcription–metabolic-flux axis. It can bind cis-elements directly or act through intermediary transcription factors; specific examples include JA-induced sakuranetin biosynthesis in rice and sesquiterpene biosynthesis in *Arabidopsis* (Hong et al., 2012; Ogawa et al., 2017). MYC2 is therefore not a pathway-specific regulator of one metabolite class but a key hub in JA-dependent defence and quality-related metabolic networks.

### 6.1 Accumulation of defensive metabolites

In *Arabidopsis*, MYC2, MYC3, and MYC4 jointly regulate glucosinolate biosynthesis. Comparative proteomics of MeJA-treated wild type and the *jin1-9/myc2* mutant identified 27 MYC2-dependent proteins. MYC2 negatively affected indolic-glucosinolate-related protein accumulation and had distinct effects on aliphatic-glucosinolate enzymes (Guo et al., 2012). Glucosinolate-biosynthetic transcripts are strongly reduced in the triple mutant, which contains very little glucosinolate and is more susceptible to generalist insects. MYC2 also functions with MYB28, MYB29, MYB34, and MYB51 in the regulation of glucosinolate-related genes (Schweizer et al., 2013). In *Arabidopsis* inflorescences, GA promotes sesquiterpene synthase expression by relieving DELLA-mediated repression of MYC2 (Hong et al., 2012). Rice OsMYC2 is required for JA-induced sakuranetin biosynthesis and interacts with MYC2-like proteins that enhance transactivation (Ogawa et al., 2017). In cotton, GhMYC2 binds G-box elements and activates the gossypol-related cytochrome P450 gene *CYP71BE79* (Han et al., 2022). Tomato MYC1 and MYC2 jointly regulate steroidal glycoalkaloids and phenylpropanoid-polyamine conjugates, contributing to inducible and constitutive chemical defence (Swinnen et al., 2022). These studies define several routes through which MYC proteins couple JA signalling to defensive-metabolite production.

### 6.2 Biosynthesis of medicinal active compounds

In *Artemisia annua*, JA-induced AaMYC2 binds G-box-like elements in the promoters of *CYP71AV1* and *DBR2* and promotes expression of key genes for artemisinin biosynthesis. *AaMYC2* overexpression increases artemisinin content, whereas RNAi-mediated suppression of *AaMYC2* reduces artemisinin accumulation and weakens the response to MeJA (Shen et al., 2016). *Taxus chinensis* TcMYC2a responds to JA signalling and upregulates genes associated with paclitaxel biosynthesis, indicating that MYC2 homologues are promising regulators of medicinal secondary metabolism (Zhang et al., 2018). In *Salvia miltiorrhiza*, *SmMYC2* overexpression increases the accumulation of water-soluble phenolic acids (Yang et al., 2017). Functional analyses further showed that SmMYC2a and SmMYC2b interact with SmJAZ1/SmJAZ2 and have related but nonredundant roles in regulating tanshinone and phenolic-acid biosynthesis (Zhou et al., 2016). In *Ginkgo biloba*, GbMYC2 promotes terpene trilactone biosynthesis by activating *GbGGPPS* expression (Zheng et al., 2024). Together, these studies reveal a degree of cross-species conservation in MYC2 regulation of terpenoid and phenolic-acid metabolism in medicinal plants. MYC2 is therefore both an upstream regulator for increasing target active compounds and a central entry point for analysing JA-induced metabolic reprogramming.

### 6.3 Tissue specificity and metabolic-flux regulation

Tobacco NtMYC2 binds nicotine-biosynthetic promoters directly and also activates NIC2-locus ERF genes, separating direct target regulation from an indirect MYC2-ERF-structural-gene cascade (Shoji and Hashimoto, 2011). In *Catharanthus roseus*, CrMYC2 activates *ORCA* transcription factors, whilst CrGBF1/2 physically dimerise with CrMYC2 and compete at shared T/G-boxes, attenuating transactivation (Zhang et al., 2011; Sui et al., 2018). In apple, MdMYC2 and *MdERF3* converge transcriptionally on alpha-farnesene biosynthesis; co-regulation does not by itself demonstrate a physical MYC2-ERF3 complex (Wang et al., 2020). In tomato, the SlMYC2-MED25 co-activator complex directly activates *SlMYB12*, forming a hierarchical transcriptional cascade that promotes fruit flavonoids (Deng et al., 2025). In lotus, the NnMYC2-NnMYB14-NnCYP80 cascade controls spatial benzylisoquinoline-alkaloid accumulation (Zhang et al., 2026). Thus, tissue specificity can arise from MYC2 abundance, partner availability, chromatin state, and the spatial expression of intermediary transcription factors and terminal enzymes.

MYC2 can drive defensive-metabolite accumulation directly, but its effects on medicinal compounds usually depend on ERF, MYB, GBF, MED25, JAZ, and other regulators. The resulting metabolic flux is strongly dependent on tissue, species, and pathway context. Constitutive MYC2 overexpression is therefore a relatively blunt engineering strategy. Tissue-specific or inducible promoters, promoter editing, and modular multigene approaches may better balance stress tolerance, product quality, and active-compound accumulation.

## 7 Genome editing and precision engineering of MYC2 networks

Genome editing can target the *MYC2* coding sequence, its regulatory regions, MYC2-binding cis-elements, or downstream regulators and susceptibility genes. Coding-sequence loss of function provides strong causal evidence but often perturbs several JA outputs simultaneously. Cis-regulatory or downstream editing may instead separate a desired stress or metabolic trait from pleiotropic growth and quality effects. **Table 2** distinguishes demonstrated applications from prospective strategies.

**Table 2:** Genome-editing strategies for MYC2 and its regulatory network.

Editing strategy	Demonstrated example	Observed outcome	Advantage	Risk/limitation	Evidence status
Direct *MYC2* knockout	*SlMYC2*; *NtMYC2a*	Reduced Botrytis or bacterial-wilt resistance; developmental/metabolic changes	Strong causal test	Broad pleiotropy; often detrimental for resistance	Validated: Shu et al., 2020; Xiao et al., 2024
Direct *MYC2* metabolic editing	*NtMYC2a* knockout	About 80% lower leaf nicotine	Large trait effect	Potential effects on nicotine-mediated herbivore defence were not directly tested	Validated: Sui et al., 2021
Functional-residue editing	OsMYC2 Ile75 deletion within the N-terminal α2 helix	Lower protein accumulation and broad JA-response defects	Maps tunable protein regions	Narrow activity window; multiple traits affected	Validated: Zu et al., 2024
Reading-frame restoration	Barley *MYC2* repair	Restored aleurone anthocyanin	Repairs an endogenous quality allele	Trait- and allele-specific; stress benefit not shown	Validated: Egorova et al., 2024
Target cis-element editing	MYC1/2-bound sterol-promoter motif in tomato	Reduced basal expression whilst retaining JA inducibility	Can decouple constitutive and induced output	Requires validated motif and promoter architecture	Validated: Swinnen et al., 2022
Downstream-node editing	*ZAT18*, *SlRR26*, *BnaA07.SUC2*	Improved bacterial, drought, or clubroot response	Preserves core *MYC2* hub; branch specificity	Target may have independent functions	Validated: Gao et al., 2022; Zhao et al., 2023; Liu et al., 2026
Promoter/base/prime editing; CRISPRa/i	No field-validated *MYC2* case identified	Designed quantitative or conditional *MYC2* output	Potential tissue and dose precision	Delivery, redundancy, off-targets, regulation, field stability	Prospective

Direct CRISPR/Cas9 editing of *MYC2* orthologues has mainly revealed pleiotropic costs. Tomato *slmyc2* mutants showed altered flower number, fruit set, and fruit shape, together with reduced defence-enzyme activity, antioxidant capacity, and MeJA-induced resistance to *Botrytis cinerea* (Shu et al., 2020). Tobacco *NtMYC2a* knockout reduced JA-induced nicotine accumulation by approximately 80% (Sui et al., 2021). A separate knockout study found narrower leaves, altered alkaloid composition, reduced resistance to *Ralstonia solanacearum*, and transcriptomic changes in immune and JA pathways (Xiao et al., 2024). In rice, a CRISPR-derived deletion of Ile75 in the N-terminal alpha2 helix lowered OsMYC2 protein accumulation. The deletion impaired root growth, senescence, spikelet development, and pathogen and herbivore resistance without abolishing DNA binding or JAZ/MED25 association (Zu et al., 2024). These experiments validate MYC2 function but caution against routine deployment of null alleles.

More selective outcomes have been obtained by repair or by editing network components. Targeted restoration of the *MYC2* reading frame in non-pigmented barley activated anthocyanin-biosynthetic genes and restored blue aleurone pigmentation, providing a demonstrated quality-trait application (Egorova et al., 2024). In tomato hairy roots, CRISPR-Cas9(VQR) editing of a MYC1/MYC2-bound cis-element in a sterol-pathway promoter reduced constitutive expression without abolishing jasmonate inducibility, illustrating how promoter editing can decouple basal and stress-induced metabolism (Swinnen et al., 2022). Editing MYC2-regulated negative or susceptibility nodes has also improved specific traits: *zat18* mutants enhance *Arabidopsis* resistance to *P. syringae*, *slrr26* mutants enhance tomato drought tolerance, and *BnaA07.SUC2* knockout enhances oilseed-rape clubroot resistance (Gao et al., 2022; Zhao et al., 2023; Liu et al., 2026). These are pathway-engineering examples, not direct *MYC2* edits, but they preserve the core hub whilst changing a defined output branch.

Prospective strategies include base or prime editing of functional residues, quantitative editing of native *MYC2* promoter motifs, stress-inducible CRISPR activation or interference, tissue-specific Cas expression, and multiplex editing of redundant MYC paralogues. Their potential advantages are allele precision, transgene-free segregants in compatible systems, and separation of constitutive growth from inducible defence. Major challenges include paralogue compensation, dosage sensitivity, off-target and structural variation, linkage of defence to yield or quality costs, genotype-by-environment effects, transformation efficiency, and jurisdiction-specific regulation. Successful deployment will require native-locus editing, multi-season field tests, and joint measurement of resistance, yield, development, metabolite composition, and non-target ecological effects; until such data exist, these strategies should be described as prospective rather than proven.

## 8 Discussion and perspectives

Current evidence supports a context-dependent model of MYC2 regulation across hormone signalling, chromatin state, protein stability, development, and metabolism. Genome-wide binding and multi-omics studies extend the MYC2 target space beyond canonical JA-defence genes, but each data layer supports a different claim. Promoter occupancy alone does not establish phenotypic causality, transcript changes may not predict protein abundance, and targeted interaction assays do not provide proteome-wide evidence. **Table 3** compares the study designs, contributions, and limitations that underpin current MYC2 models.

**Table 3:** Comparative multi-omics evidence and research methods used to define MYC2 networks.

Study/system	Data layers	Design	Main analytical contribution	Key limitation	Reference
*Arabidopsis myc2*	Transcriptome	WT vs *myc2* after JA; downstream mutant tests	Revealed positive and negative JA branches and intermediary TFs	Expression changes include indirect effects	Dombrecht et al., 2007
*Arabidopsis* MYC2/3/4	ChIP-seq, transcriptome, hormones	Water-spray time course; WT vs *myc234*	Resolved a rapid clade-dependent TF network and JA amplification	Clade-level effects cannot be assigned to MYC2 alone	Van Moerkercke et al., 2019
*Arabidopsis* JA response	Time-series RNA-seq, chromatin, proteome, phosphoproteome	Integrated network modelling plus mutant validation	Linked transcription, splicing, protein abundance, phosphorylation, and chromatin	JA-system framework; not all nodes are MYC2 specific	Zander et al., 2020
*Arabidopsis* WT vs *jin1-9*	Comparative proteome	2D gels and MALDI-TOF/TOF after MeJA	Identified MYC2-dependent metabolic and oxidative-stress proteins	Limited proteome coverage and temporal resolution	Guo et al., 2012
*Arabidopsis* WT vs *myc234*	Targeted protein turnover, mRNA, hormones	Heavy-N labelling and PRM LC-MS during PTI recovery	Showed post-transcriptional control of growth-defence transitions	Targeted panel; clade-level attribution	Abukhalaf et al., 2023
Maize ZmMYC2	DAP-seq plus RNA-seq	Overlap of *in vitro* binding and expression response	Generated a broad candidate direct-target atlas	DAP-seq is not *in vivo* occupancy; functional validation is limited	Liu et al., 2024
Rice OsMYC2	Time/tissue RNA-seq, ChIP-seq, metabolomics	Three tissues, five time points, herbivore datasets, genetics	Separated core, tissue-specific, and negative-feedback sectors	No matched quantitative proteome; constitutive tagged line used for ChIP	Chen et al., 2024
Poplar MYC2 paralogues	Root transcriptome, DAP-seq, metabolites	OE and silencing during ectomycorrhizal colonisation	Connected paralogue dosage, terpene defence, and symbiosis	Direct and indirect targets require further separation	Marqués-Gálvez et al., 2024
Tobacco NtMYC2a	RNA-seq plus chemical and disease phenotyping	CRISPR knockout vs WT	Linked MYC2 loss to immune, JA, growth, and alkaloid changes	Single genotype/stress context; direct targets unresolved	Xiao et al., 2024
MYC2 protein regulation	Targeted protein assays	Phosphorylation, stability, PPI, reporters	Defined MPK6- and JMJC3-dependent regulation	Targeted evidence, not unbiased proteomics	Im et al., 2025; Xu et al., 2026

ChIP-seq measures *in vivo* chromatin occupancy; DAP-seq measures *in vitro* DNA binding; RNA-seq measures transcript abundance; proteomics measures protein abundance or turnover; targeted PPI and immunoblot assays test selected proteins and should not be labelled global proteomics. WT, wild type; PTI, pattern-triggered immunity; PRM, parallel reaction monitoring; OE, overexpression.

Cross-species translation requires conservation and divergence to be tested separately. Rice has a major MYC2-type factor and tissue-specific defence sectors, whereas *Arabidopsis* often requires MYC2/MYC3/MYC4 higher-order mutants. Crop and medicinal species may also contain duplicated MYC2-like genes with overlapping or non-interchangeable functions. Drought-associated phenotypes in tomato, sorghum, tea, melon, and poplar arise through distinct target modules. By contrast, *Arabidopsis* salt sensitivity involves *CAT2* repression, and pathogen outcomes vary with JA-SA antagonism. These differences delimit the conditions under which MYC2-directed engineering may succeed.

Application evidence spans distinct levels of maturity. Direct *MYC2* manipulation has demonstrated large effects on defence, development, and specialised metabolism, but null mutations often produce undesirable pleiotropy. Editing a validated MYC2-bound cis-element or a branch-specific downstream regulator has produced more selective effects in experimental systems. Tissue-specific CRISPRa/i, quantitative promoter editing, base or prime editing, and multiplex paralogue tuning remain prospective. Constitutive overexpression also requires caution because gains in resistance or metabolites may be offset by reduced growth, fertility, yield, or quality. Useful MYC2 engineering will therefore require control of allele, tissue, dose, and timing rather than maximal activity.

MYC2 connects JA perception with transcription, growth, defence, and specialised metabolism. Interpreting this network requires physical interaction, promoter binding, transcriptional regulation, and pathway association to be distinguished explicitly. Many *Arabidopsis* outputs are shared with MYC3 and MYC4, whereas crop orthologues differ in copy number, partners, tissues, and targets. Across species, beneficial and detrimental phenotypes depend on attacker lifestyle, hormone antagonism, target-gene polarity, ROS localisation, developmental timing, and perturbation design. Direct *MYC2* editing is feasible but often pleiotropic; promoter, cis-element, or downstream-node editing currently offers a more selective route. Conditional CRISPR and precision allele tuning remain prospective. Future studies should combine native-locus genetics, tissue-resolved multi-omics, quantitative proteomics, and realistic combined-stress field trials. MYC2 networks will become credible breeding targets only when resistance gains are evaluated alongside yield, development, metabolite composition, and ecological effects.
